# Trajectories of Gender Identity and Depressive Symptoms in Youths

**DOI:** 10.1001/jamanetworkopen.2024.11322

**Published:** 2024-05-22

**Authors:** André Gonzales Real, Maria Inês Rodrigues Lobato, Stephen T. Russell

**Affiliations:** 1 Department of Human Development and Family Sciences, The University of Texas at Austin; 2 Department of Psychiatry, Hospital de Clínicas de Porto Alegre, Universidade Federal do Rio Grande do Sul, Porto Alegre, Brazil

## Abstract

**Importance:**

Concerns about the mental health of youths going through gender identity transitions have received increased attention. There is a need for empirical evidence to understand how transitions in self-reported gender identity are associated with mental health.

**Objective:**

To examine whether and how often youths changed self-reported gender identities in a longitudinal sample of sexual and gender minority (SGM) youths, and whether trajectories of gender identity were associated with depressive symptoms.

**Design, Setting, and Participants:**

This cohort study used data from 4 waves (every 9 months) of a longitudinal community-based study collected in 2 large cities in the US (1 in the Northeast and 1 in the Southwest) between November 2011 and June 2015. Eligible participants included youths who self-identified as SGM from community-based agencies and college groups for SGM youths. Data analysis occurred from September 2022 to June 2023.

**Exposure:**

Gender identity trajectories and gender identity variability.

**Main Outcomes and Measures:**

The Beck Depression Inventory for Youth (BDI-Y) assessed depressive symptoms. Gender identity variability was measured as the number of times participants’ gender identity changed. Hierarchical linear models investigated gender identity trajectories and whether gender identity variability was associated with depressive symptoms over time.

**Results:**

Among the 366 SGM youths included in the study (mean [SD] age, 18.61 [1.71] years; 181 [49.4%] assigned male at birth and 185 [50.6%] assigned female at birth), 4 gender identity trajectory groups were identified: (1) cisgender across all waves (274 participants ), (2) transgender or gender diverse (TGD) across all waves (32 participants), (3) initially cisgender but TGD by wave 4 (ie, cisgender to TGD [28 participants]), and (4) initially TGD but cisgender by wave 4 (ie, TGD to cisgender [32 participants]). One in 5 youths (18.3%) reported a different gender identity over a period of approximately 3.5 years; 28 youths varied gender identity more than twice. The cisgender to TGD group reported higher levels of depression compared with the cisgender group at baseline (*Β* = 4.66; SE = 2.10; *P* = .03), but there was no statistical difference once exposure to lesbian, gay, bisexual, and transgender violence was taken into account (*Β* = 3.31; SE = 2.36; *P* = .16). Gender identity variability was not associated with within-person change in depressive symptoms (*Β* = 0.23; SE = 0.74; *P* = .75) or the level of depressive symptoms (*Β* = 2.43; SE = 2.51; *P* = .33).

**Conclusions:**

These findings suggest that gender identity can evolve among SGM youths across time and that changes in gender identity are not associated with changes in depressive symptoms. Further longitudinal work should explore gender identity variability and adolescent and adult health.

## Key Points

**Question:**

Are gender identity trajectories and changes in youth-reported gender identity associated with depressive symptoms over time?

**Findings:**

In this cohort study involving 366 sexual and/or gender minority youths (aged 15-21 years), 1 in 5 (18.2%) reported a different gender identity over time. Youths transitioning to a transgender or gender diverse identity reported higher levels of depressive symptoms at baseline; depressive symptoms disparities were explained by exposure to lesbian, gay, bisexual, and transgender violence, but frequency of gender identity variability was not associated with the level or changes in depressive symptoms.

**Meaning:**

In this study, changes in gender identity were not associated with depressive symptoms, suggesting that gender identity exploration is a normal part of adolescent development for some youths.

## Teaser

This cohort study explores the frequency of self-reported changes in gender identity among youths and whether trajectories of gender identity were associated with depressive symptoms.

## Introduction

Gender identity refers to one’s inner sense of being a man, a woman, or something else.^1^ Transgender and gender diverse (TGD) people are those for whom gender identity does not align with societal expectations based on their sex assigned at birth. Recent studies^2,3^ show that 1.4% to 1.8% of US youths identify as TGD.

For most people, gender identity is a stable aspect of the self^4,5^; development or change in gender identity over time is less understood. Several studies^6,7,8,9^ have explored whether children who met criteria for gender identity disorder (GID; an obsolete diagnostic criteria used in the *Diagnostic and Statistical Manual of Mental Disorders* [Fourth Edition]) in childhood still meet GID criteria in adolescence or adulthood. Approximately 85% of the children in these studies came to identify as a sexual minority without GID in adolescence or adulthood.^10^ These studies refer to persistence and desistance of gender incongruence. However, interpretation from these studies are limited because a substantial proportion of study participants were subthreshold for GID diagnosis in childhood,^11^ and using such outdated diagnostic criteria is problematic because one could meet GID in childhood criteria without necessarily identifying as TGD.^12^ Nonetheless, recent work^13,14^ indicates that a large majority of socially transitioned TGD children still identified as TGD 2.5 to 5 years later. Similarly, a UK study^15^ found that among TGD youths, 91.7% continuously identified as TGD while being followed up by a gender clinic for minors.

For some, however, understanding of one’s gender may vary over time.^16^ Some scholars refer to this variability as dynamic gender presentations,^16^ gender journeys,^17^ retransition, or detransition,^18^ although there is no consensus on definitions of detransition.^19^ Of note, Olson et al^13^ found a 7.3% retransition rate among socially transitioned TGD children, indicating retransitions are not common in this group. Among adolescents and adults, it is estimated that 1% to 13% may experience retransition or detransition.^18,19,20,21^ A recent longitudinal study^22^ using a national probability sample found that approximately 1% of the US population reported different gender identities over a period of 4 years, a pattern more prevalent in younger than older generations. Importantly, among TGD people who reported past detransitions, external factors such as social rejection are often reported as reasons associated with detransitioning.^18^ Little is known about gender identity variability among adolescents and young adults.

Recently, a controversial theory of rapid onset gender dysphoria (ROGD) was developed based on a survey of parents of TGD youths who transitioned in adolescence.^23^ In the ROGD framework, gender dysphoria experienced by adolescents and young adults without prior indicators would be due to social contagion and compromised mental health and would disproportionately affect youths assigned female at birth. These youths would also experience a decline in mental health and functioning after transitioning.^23^ The original publication has been criticized because of biased sampling and misleading interpretation^24,25,26^; furthermore, a correction of the original work by Littman et al^23^ emphasized that ROGD is not a formal diagnosis.^27^ Recent cross-sectional studies^28,29,30^ do not support the ROGD hypothesis. Longitudinal, community-based studies are needed to investigate whether people who transition to a TGD identity in adolescence report compromised mental health before gender identity transition or worsening mental health after. The only study,^22^ to our knowledge, which explores how gender identity variability may affect health found that individuals whose gender identity varied over time engaged in more health-related risk behavior.

The current study assesses trajectories of gender identity in youths from a community-based sample, aged 15 to 21 years, over 4 assessment points, examining variability or changes in gender identity over time. Given that TGD youths often seek hormone therapy to treat gender dysphoria^31,32^ and because exposure to lesbian, gay, bisexual, and transgender (LGBT) violence is associated with the mental health of TGD youths,^33,34^ our analyses account for hormone and puberty blocker use and exposure to LGBT violence. Analyses investigated whether depressive symptoms varied across distinct trajectories of gender identity among youths, and associations of frequency of gender identity variability with depressive symptoms over time.

## Methods

### Participants and Procedures

This cohort study was approved by the institutional review boards of New York University and the University of Arizona and followed the Strengthening the Reporting of Observational Studies in Epidemiology (STROBE) reporting guideline. Data come from a community-based longitudinal study of sexual and/or gender minority (SGM) youths between the ages of 15 and 21 years at baseline (4 waves of data collection, every 9 months [2012-2015]).^35^ See the flowchart of participants included in the study across waves in the eFigure in Supplement 1. Community leaders recruited youths who identified as SGM from community-based agencies and college groups for SGM youths. Recruitment also occurred through referrals from other participants. Data were collected in 2 large cities in the US (1 in the Northeast and 1 in the Southwest) (eAppendix 1 in Supplement 1). Parental consent was waived for participants younger than 18 years to assure safety for youths who were not out to their parents; an independent representative was present to ensure youths participants’ assent. Those older than 18 years provided written informed consent. Participants received financial compensation. To capture gender identity variability, we focused on youths who participated in at least 3 waves of the study. At each wave, participants were coded as TGD when their gender identity did not match their sex assigned at birth.

### Measures

#### Reported Gender Identity, Gender Identity Change, and Trajectories

A 2-step approach was used to assess youths gender identity.^36^ First, sex at birth was assessed at wave 1 (male and female). Second, at waves 1 to 4, participants were asked, “What is your Gender Identity?” (response options included “man,” “woman,” “genderqueer,” “trans-woman,” “trans-man,” and write-in). Write-in responses were coded as either cisgender, binary transgender (ie, transgender woman or transgender man), or genderqueer and nonbinary. Examples of write-in responses included “woman, queer,” “gender non-conforming,” and “gender-fluid.” At each wave, the 2 measures were paired, and participants were categorized in 1 of 3 gender identities: (1) binary transgender, (2) genderqueer and nonbinary, or (3) cisgender.

A variable indicating the gender identity variability compared with the previous wave was generated for waves 2 to 4. Frequency of gender identity variability was measured as the number of times participants’ gender identity changed across the 4 waves of the study (from 0 to 3).

#### Depressive Symptoms

Depressive symptoms were measured utilizing the Beck Depression Inventory for Youth (BDI-Y), which assesses negative thoughts, sadness, and depressive symptoms.^37^ A sum score was calculated, with higher scores indicating more depressive symptomatology (average internal consistency across waves of α = .94). Sum scores of 13 or less are considered normal; scores of 14 or greater may indicate mild to severe depressive symptoms.

### Covariates

Based on prior research, demographic characteristics described below were included in the adjusted models. They were included as possible confounders between factors and the outcome.^38,39,40,41,42^

#### Demographic Characteristics

Time invariant demographic variables were collected at wave 1 and included age at baseline, sex assigned at birth (0 = male; 1 = female), receipt of free lunch in high school (0 = no; 1 = yes; 2 = not reported), and race and ethnicity (0 = non-Latino White; 1 = non-Latino Black; 2 = Latino; 3 = other race or ethnicity or did not report), and recruitment site (0 = Southwest; 1 = Northeast). The other race and ethnicity included Asian, Pacific Islander, American Indian or Alaska Native, and multiracial individuals, and was created due to small sample size of each of the individual categories. Race and ethnicity were included in the study to account for health disparities that may be associated with social determinants and societal marginalization. See Appendix 2 in Supplement 1 for detailed information on the measures utilized for this study.

#### Explanatory Variables

Given that youths identifying as TGD tend to report more exposure to LGBT violence^43^ and often seek puberty blockers and hormone therapy to reduce gender dysphoria^34,44^ and that these factors are known to be associated with mental health,^31,33,34^ these measures were included in final models as possible explanatory variables between transition and depression. Alternatively, exposure to LGBT violence could be a confounding variable between gender identity changes and depressive symptoms; prior work has suggested that TGD individuals may detransition as a response to stigma,^18^ and exposure to LGBT violence is also associated with more depressive symptoms.^33^

#### Hormone Therapy and Puberty Blocker Use

At waves 2 to 4, participants reported history of hormone therapy and puberty blocker use. Participants were asked, “Have you ever taken (a) hormone replacement therapy? or (b) puberty blockers?” (0 = no; 1 = yes).

#### Cumulative Exposure to Violence Due to LGBT Identity

Exposure to violence due to LGBT identity was assessed using a 6-item scale in which participants reported how often they had experienced different forms of LGBT violence (0 = never; 3 = at least 3 times).^45^ At wave 1, participants were asked to consider these experiences in their lifetime. In subsequent waves, participants were asked to consider only the past 9 months. At each wave, a mean score was computed. To obtain a cumulative score, the sum score was calculated by adding the scores from the previous waves to each measure of exposure to LGBT violence across time.

### Statistical Analysis

Data were managed and analyzed using Stata 18.0 (StataCorp). First, we conducted analyses of variance to test group differences in frequency of gender identity variability. Bonferroni adjustments were applied to adjust for multiple group comparisons (significance for these analyses were set at *P* < .008). Hierarchical level modeling (HLM) was used to analyze trajectories of depressive symptoms. To estimate within- and between-person effects, time-varying factors (ie, cumulative exposure to LGBT violence and frequency of gender identity variability) were decomposed into 2 components; between-person (BP) components (level 2) are assessed by the person mean across waves; and within-person (WP) components (level 1) are assessed by the individual deviation from their own mean across waves.^46^ Thus, the BP component contrasted depressive symptoms of youths who had more gender identity variability with other youths who had less or no gender identity variability, whereas the WP component contrasted depressive symptoms when a participant had more gender identity variability with other periods in which the same participant had less or no gender identity variability.

An empty mean model (ie, without factors included) was tested to estimate the degree to which depressive symptoms variation was associated with BP factors (intraclass correlation [ICC]). Unadjusted and adjusted models examined whether the trajectory of depressive symptoms varied across gender identity trajectory groups (model 1 and model 2, respectively). Based on model 2, model 3 investigated whether the frequency of gender identity variability was associated with depressive symptoms in both BP and WP levels, while also accounting for hormone and puberty blocker use and cumulative exposure to LGBT violence. Significance testing for HLM analyses were set at a 2-tailed *P* < .05. Given that missingness in key variables was substantively low (<3%), we addressed missing values with listwise deletion (at the waves participants had missing values) due to the low impact in the sample size (see the eTable in Supplement 1 for sensitivity analysis). HLM uses a mixed-effects model that works with all data available in longitudinal analyses. Data analysis was conducted from September 2022 to June 2023.

## Results

### Descriptive Statistics

The analytic sample included 366 SGM youths (mean [SD] age, 18.61 [1.71] years; 181 [49.4%] assigned male at birth and 185 [50.6%] assigned female at birth; 149 Latino [40.7%]; 84 non-Latino Black [23.0%]; 75 non-Latino White [20.5%]; 58 [18.9%] other race or ethnicity or not reported) of whom 274 (74.9%) identified as cisgender at all waves and 92 (25.1%) identified as TGD at some point in the study. Descriptive statistics of the study participants are described in Table 1. The majority of participants were recruited in the Northeast site (254 participants [69.4%]) and 196 participants (53.6%) had received free lunch in high school, indicating possible lower socioeconomic status. Trajectory patterns were categorized into 4 groups, including participants who were (1) cisgender across all waves (274 participants), (2) TGD (including binary transgender and genderqueer or nonbinary) across all waves (32 participants), (3) cisgender at wave 1 or 2 but by wave 4 identified as TGD (ie, cisgender to TGD [28 participants; 26 of these participants (92.9%) identified as cisgender at wave 1]), and (4) TGD at any wave but by wave 4 identified as cisgender (ie, TGD to cisgender [32 participants]). Overall, 1 in 5 participants (18.3%) reported a different gender identity over the study period.

**Table 1.** Demographic Characteristics of the Baseline Among Different Gender Identity Trajectories Membership

Demographic	Participants, No. (%)				
	Full sample (N = 366)^a^	Cisgender (n = 274)^b^	TGD (n = 32)^b^	Cisgender to TGD (n = 28)^b^	TGD to cisgender (n = 32)^b^
Sex at birth					
Male	181 (49.4)	145 (80.1)	13 (7.2)	12 (6.6)	11 (6.1)
Female	185 (50.6)	129 (69.7)	19 (10.3)	16 (8.7)	21 (11.4)
Age, mean (SD) y	18.61 (1.71)	18.65 (1.72)	18.56 (1.58)	18.79 (1.47)	18.2 (1.98)
Race and ethnicity					
Latino	149 (40.7)	111 (74.5)	10 (6.7)	12 (6.7)	16 (10.7)
Non-Latino Black	84 (23.0)	69 (82.1)	5 (6.0)	4 (4.8)	6 (7.1)
Non-Latino White	75 (20.5)	55 (73.3)	13 (17.3)	3 (4.0)	4 (5.3)
Other or not reported^c^	58 (15.9)	39 (67.2)	4 (6.9)	9 (6.9)	6 (10.3)
Site					
Northeast	254 (69.4)	195 (76.8)	20 (7.9)	19 (7.5)	20 (7.9)
Southwest	112 (30.6)	79 (70.5)	12 (10.7)	9 (8.0)	12 (10.7)
Received free lunch					
No	168 (43.8)	120 (71.4)	18 (10.7)	18 (10.7)	12 (7.1)
Yes	196 (53.6)	154 (78.6)	14 (7.1)	8 (4.1)	20 (10.2)
Not reported	2 (0.6)	0	0	2 (100)	0
Hormone use					
No	339 (92.6)	274 (80.8)	12 (3.5)	22 (6.5)	31 (9.1)
Yes	27 (7.4)	0 (0.0)	20 (74.1)	6 (22.2)	1 (3.7)
Puberty blocker use					
No	354 (96.7)	274 (77.4)	23 (6.5)	25 (7.1)	32 (9.0)
Yes	12 (3.3)	0	9 (75.0)	3 (25.0)	0
Frequency of gender identity variability					
0	299 (81.7)	274 (91.6)	25 (8.4)	0	0
1	39 (10.7)	0	5 (12.8)	21 (53.9)	13 (33.3)
2	21 (5.7)	0	1 (4.8)	4 (19.1)	16 (76.2)
3	7 (1.9)	0	1 (14.3)	3 (42.9)	3 (42.9)

Abbreviation: TGD, transgender and gender diverse.

^a^ Percentages by column.

^b^ Percentages by row.

^c^ Other or not reported included those who did not report any race or ethnicity and participants who were American Indian or Alaska Native, Asian, Pacific Islander, or multiracial. These participants were grouped in this category due to small numbers.

While 20 of 32 participants (62.5%) in the TGD group reported hormone use, only 6 of 28 participants (21.4%) in the cisgender to TGD group, and 1 of 32 participants (3.1%) in the TGD to cisgender group had used hormones. Use of puberty blockers was reported by 12 of the 92 participants who identified as noncisgender (ie, binary transgender or genderqueer and nonbinary) during the study; the majority were from the TGD group. Table 2 displays cumulative exposure to LGBT violence scores for the 4 gender identity trajectory groups. Participants presented mild levels of depression across all waves (mean [SD], 13.61 [11.02]).^37^

**Table 2.** Descriptive Statistics of Cumulative Exposure to LGBT Violence Across Waves

Wave	Cumulative exposure to LGBT violence, mean (SD)				
	Full sample (N = 366)	Cisgender (n = 274)	TGD (n = 32)	Cisgender to TGD (n = 28)	TGD to cisgender (n = 32)
1	0.63 (0.67)	0.58 (0.65)	0.84 (0.63)	0.89 (0.80)	0.66 (0.67)
2	0.87 (0.88)	0.77 (0.82)	1.18 (0.75)	1.29 (1.10)	0.99 (1.05)
3	1.04 (1.00)	0.93 (0.95)	1.43 (0.96)	1.52 (1.19)	1.16 (1.11)
4	1.18 (1.18)	1.04 (1.10)	1.61 (1.08)	1.81 (1.40)	1.41 (1.44)

Abbreviations: LGBT, lesbian, gay, bisexual, and transgender; TGD, transgender and gender diverse.

**Figure 1.**
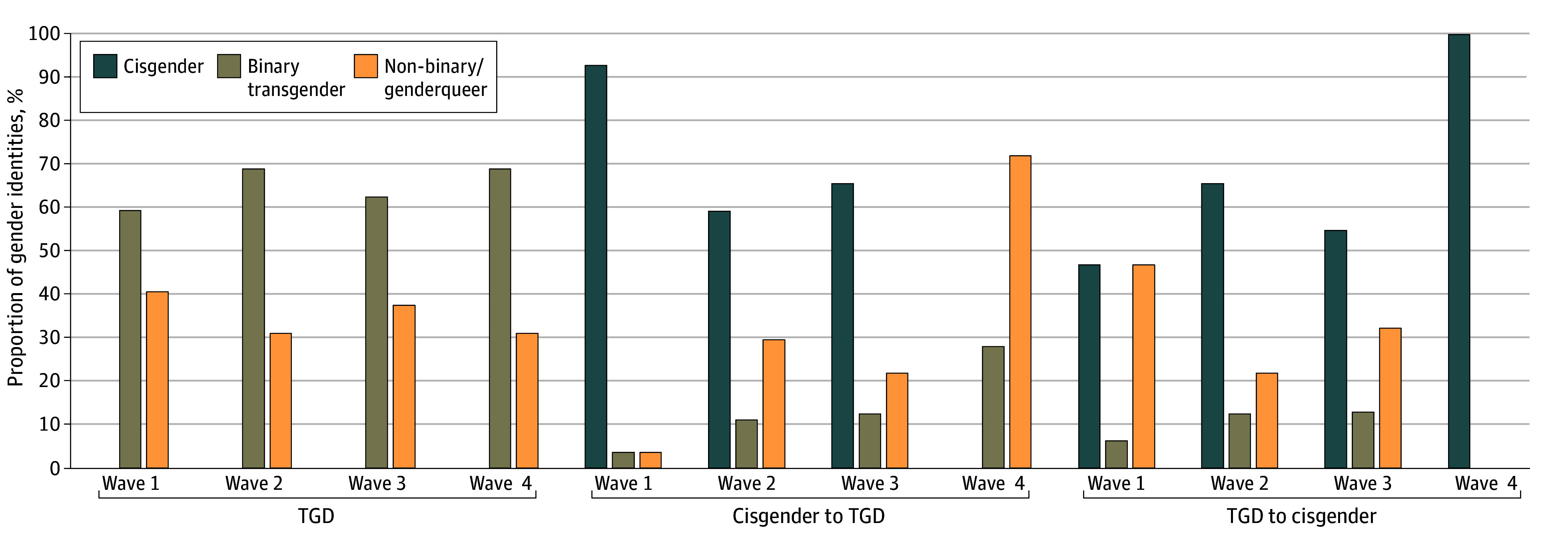
Proportions of Gender Identities by Gender Identity Trajectory Groups Across Waves TGD indicates transgender and gender diverse.

Figure 1 displays the proportion of gender identities for each trajectory group across waves. For participants from the cisgender to TGD and TGD to cisgender groups (ie, the groups defined by gender identity change), when they identified as TGD, they most often identified as genderqueer or nonbinary. The TGD group, nonetheless, most often identified as binary transgender (ie, transgender woman or transgender man). Changing gender identities at least twice was relatively common among noncisgender youths during the study (28 of 92 participants [30.4%]). The majority of youths in the TGD to cisgender group (19 of 32 participants [59.4%]) reported a different gender identity at least twice across waves. Youths in the TGD group reported fewer gender identity changes than the other 2 groups, but the TGD to cisgender and cisgender to TGD groups did not differ from one another.

### Longitudinal Analyses

**Figure 2.**
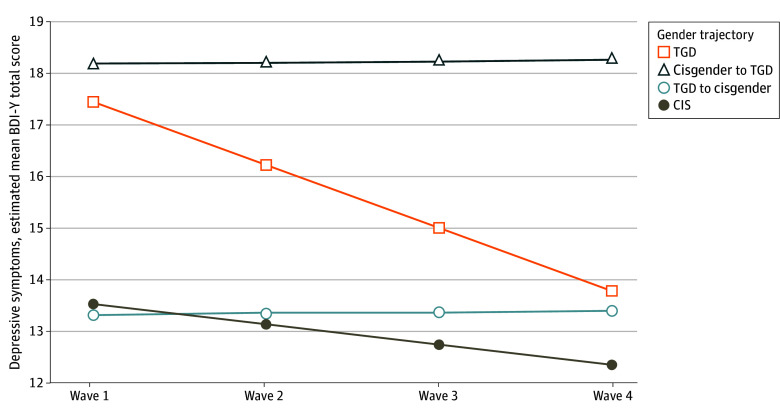
Trajectories of Depressive Symptoms Over Time Among Youths With Different Gender Identity Trajectories The figure shows estimated means for the Beck Depression Inventory for Youths (BDI-Y) total score at each wave. CIS indicates cisgender; TGD, transgender and gender diverse.

The ICC results showed 58.5% of the variance of depressive symptoms was at the BP level, and 41.5% was at the WP level (ICC coefficient, 0.585; 95% CI, 0.536 to 0.633). HLM unconditional models indicated that depressive symptoms had a linear trajectory accounting for random effects. HLM analyses assessing trajectories of depressive symptoms show that at baseline (intercepts) in the unadjusted model (model 1), participants from the TGD and the cisgender to TGD groups reported higher levels of depression when compared with the cisgender group (*Β* = 3.91; SE = 1.98; *P* = .048; vs *Β* = 4.66; SE = 2.10; *P* = .03) (Table 3 and Figure 2). However, in the model adjusted for demographic characteristics (model 2), only the cisgender to TGD group statistically differed from the cisgender group (*Β* = 4.82; SE = 2.10; *P* = .02). In this model, post hoc group comparisons indicated that the cisgender to TGD group also reported more depressive symptoms at baseline when compared with the TGD to cisgender group, but this finding was not significant (*Β* = 6.02; SE = 2.30; *P* = .05). There were no gender identity trajectory group differences in the rate of change of depressive symptoms over time (slopes), and there were no differences based on post hoc group comparisons. Importantly, the baseline difference in depressive symptoms between cisgender to TGD group and cisgender group was not significant after accounting for exposure to LGBT violence (model 3, *Β* = 3.31; SE = 2.36; *P* = .16).

**Table 3.** Multivariate Hierarchic Linear Model of Depressive Symptoms (N = 366)

Measurement	Model 1		Model 2		Model 3	
	*Β* (SE)	*P* value	*Β* (SE)	*P* value	*Β* (SE)	*P* value
Fixed effects						
Within-person (level 1)						
Linear time change^a^	−0.39 (0.24)	.10	−0.39 (0.24)	.10	−0.65 (0.26)	.01
Cumulative victimization	NA	NA	NA	NA	1.52 (0.69)	.03
Frequency of gender identity variability	NA	NA	NA	NA	0.23 (0.74)	.75
Between-person (level 2)						
Gender identity trajectory						
Cisgender	0 [Reference]	NA	0 [Reference]	NA	0 [Reference]	NA
TGD	3.91 (1.98)	.048	2.72 (1.92)	.16	3.34 (2.38)	.16
Cisgender to TGD	4.66 (2.10)	.03	4.82 (2.10)	.02	3.31 (2.36)	.16
TGD to cisgender	−0.21 (1.98)	.92	−1.20 (1.91)	.53	−3.02 (2.48)	.22
Time × gender identity trajectory^a^						
Linear time change × cisgender	0 [Reference]	NA	0 [Reference]	NA	0 [Reference]	NA
Linear time change x TGD	−0.83 (0.73)	.26	−0.83 (0.73)	.26	−0.97 (0.73)	.19
Linear time change x cisgender to TGD	0.43 (0.78)	.58	0.43 (0.78)	.58	0.17 (0.80)	.84
Linear time change x TGD to cisgender	0.42 (0.74)	.57	0.43 (0.74)	.56	0.23 (0.78)	.77
Receipt of free lunch						
No	0 [Reference]	NA	0 [Reference]	NA	0 [Reference]	NA
Yes	NA	NA	−0.18 (0.99)	.85	−0.59 (0.96)	.54
Not reported	NA	NA	−9.25 (6.36)	.15	−10.83 (6.36)	.09
Age (centered at the mean)	NA	NA	−0.39 (0.28)	.17	−0.50 (0.27)	.07
Sex assigned at birth						
Male	0 [Reference]	NA	0 [Reference]	NA	0 [Reference]	NA
Female	NA	NA	3.06 (0.93)	.001	3.84 (0.93)	<.001
Recruitment site						
Northeast	0 [Reference]	NA	0 [Reference]	NA	0 [Reference]	NA
Southwest	NA	NA	2.23 (1.04)	.03	2.47 (1.01)	.01
Race and ethnicity						
Non-Latino White	0 [Reference]	NA	0 [Reference]	NA	0 [Reference]	NA
Non-Latino Black	NA	NA	−4.27 (1.48)	.004	−3.86 (1.44)	.01
Latino	NA	NA	−1.45 (1.30)	.27	−1.68 (1.26)	.18
Other or not reported	NA	NA	−1.76 (1.55)	.26	−1.90 (1.50)	.21
Hormone use						
No	0 [Reference]	NA	0 [Reference]	NA	0 [Reference]	NA
Yes	NA	NA	NA	NA	−4.72 (2.74)	.08
Puberty blocker use	NA	NA	NA	NA		
No	0 [Reference]	NA	0 [Reference]	NA	0 [Reference]	NA
Yes	NA	NA	NA	NA	3.54 (3.37)	.29
Cumulative exposure to LGBT violence	NA	NA	NA	NA	2.59 (0.51)	<.001
Frequency of gender identity variability	NA	NA	NA	NA	2.43 (2.51)	.33
Intercept	13.54 (0.64)	NA	13.52 (1.31)	NA	11.56 (1.36)	NA
Random effects						
Time variance	6.68 (1.22)	NA	6.69 (1.22)	NA	6.66 (1.23)	NA
Intercept variance	83.85 (8.43)	NA	75.18 (7.84)	NA	68.93 (7.41)	NA
Time and intercept covariance	−9.23 (2.56)	NA	−8.80 (2.49)	NA	−8.27 (2.44)	NA
Residual variance	39.19 (2.14)	NA	39.19 (2.14)	NA	38.84 (2.14)	NA

Abbreviations: LGBT, lesbian, gay, bisexual, and transgender; NA, not applicable; TGD, transgender and gender diverse.

^a^ Slope of depressive symptoms for the cisgender group.

Lastly, tests of the association of gender identity variability with depressive symptoms over time (model 3) showed that, at the BP level, patterns of depressive symptoms did not differ for youths who reported more variability in gender identity compared with their counterparts who reported less or no gender identity variability (*Β* = 2.43; SE = 2.51; *P* = .33). Furthermore, WP estimates indicated that youths did not report more depressive symptoms at times when they reported more gender identity variability compared with other periods when they reported less or no gender identity variability (*Β* = 0.23; SE = 0.74; *P* = .75).

## Discussion

Gender identity is complex, and typically characterized as stable, or as a movement from cisgender to TGD (and for some, back to cisgender). Even longitudinal studies typically ask participants about their gender identity only once, overlooking ways that understanding of gender identity may vary or change for youths over time. Although a few longitudinal studies^13,14^ have examined whether gender identity varies among socially transitioned TGD children, trajectories of gender identity in samples of youths are still not well understood.^47,48^ In this community-based longitudinal cohort study, 1 in 5 (18.3%) youths reported a different gender identity over a period of approximately 3.5 years (the majority reported the same gender identity over time). Almost one-third of the youths who reported a different gender identity did so more than twice. These findings differ from clinical samples where the majority of TGD youths consistently identified as TGD,^15^ but complement recent longitudinal work^48^ revealing that while gender identity is stable for the majority of youths, shifts in gender identity are not uncommon and should not be considered abnormal. Our findings empirically support the idea that gender identity can be fluid or in development for some youths.^11,17,48,49,50,51^ Importantly, while changes in gender identities can be driven by developmental gender identity exploration,^52^ prior work has indicated that it can be driven by a social adaptation to stigma.^18^

Much scientific and public attention has focused on mental health for TGD youths; our findings show that youths who reported the most change in gender identities during the study period (TGD to cisgender) were in one of the groups with the lowest levels of depression across all waves. It is possible that positive mental health can help youths feel comfortable exploring gender identity, despite societal stigma. Notably, depressive symptoms among participants in this group were stable over time. This stability might be a result of floor effects. Also, for this group, identity variability was typically between nonbinary and cisgender identities; they may have had less nonconforming gender expressions, which perhaps relates to their lower exposure to LGBT violence relative to other TGD groups. Furthermore, gender identity variability was not associated with more depressive symptoms, either between participants (BP) or for individuals over time (WP). Thus, youths who reported more changes in their gender identities were no different in terms of their mental health compared with those with fewer changes, and longitudinally, youths were not more depressed after a shift in their gender identities. These findings are more consistent with an explanation of adolescent gender identity exploration and development, rather than arguments that gender identity changes would be associated with compromised or worsening in mental health.^11^

Our examination of groups based on gender identity trajectories (consistently cisgender or TGD, or reported changes to or from cisgender and TGD) showed that youths who reported the most change in gender identities over time (the cisgender to TGD and TGD to cisgender groups) tended to often identify as genderqueer or nonbinary, consistent with prior studies that show that youths more often identify with nonbinary gender identities.^29,48,53^ It may be that youths who identify as genderqueer or nonbinary may be more comfortable with gender identity exploration.

### Implications for Current Controversies

Concerns about youths who identify as TGD have been raised in the past decade, particularly due to reports of greater numbers seeking treatment,^54^ in particular by youths assigned female at birth.^47,54,55^ Results from this study offer insight into several debates.

ROGD proponents suggest that the rising numbers of TGD-identifying youths are due to compromised mental health and social contagion.^23^ Results of recent studies^28,29^ are not consistent with these claims. Psychological well-being and demographic characteristics of youths referred to transgender clinics have been mostly consistent for more than a decade (except for sex ratio).^55^ Furthermore, in a cross-sectional study,^28^ later transgender identity acknowledgment was not associated with more compromised mental health among TGD youths. Findings from our study are also inconsistent with the ROGD hypothesis in at least 2 ways. First, although youths whose identities changed from cisgender to TGD reported higher levels of depressive symptoms when compared with consistently cisgender youths, these differences disappeared when we accounted for exposure to LGBT violence. Of note, they also experienced more exposure to LGBT violence than youths who identified consistently as cisgender, possibly due to higher gender nonconformity.^56^ Second, our study indicates that youths who transitioned to TGD during the study (cisgender to TGD) experienced stable levels of depressive symptoms over time, a marked contrast with the argument that youths who identify as TGD in adolescence and early adulthood will experience worsening mental health.^23,24^ Yet, despite being stable, youths from the cisgender to TGD group reported sustained high levels of depressive symptoms over time. In addition to the accumulation of exposure to LGBT violence among this group (Table 2), transitioning to a more stigmatized identity may be mentally taxing because of exposure to new types of violence^57^ (eg, gender-based violence), expectation of rejection from family and others,^1,45^ and loss of support.

### Limitations

Despite substantial strengths of this study, including its community-based, longitudinal design, there are several limitations. While solely relying on self-reported gender identity, we were unable to examine, for example, which participants met the criteria for gender dysphoria. However, scholars have pointed out the importance of having youths report their own gender,^12^ including providing not only binary options.^58^ Furthermore, youths were not asked to explain variation in their gender identities. While transitions are part of developmental gender identity exploration for many youths,^52^ prior work has shown that external pressures are often associated with detransitioning from a TGD identity.^18,59^ We also do not know what proportion of these TGD youths went through social transitions (ie, changed pronouns or gender presentation to align with experienced gender); TGD youths may be more targeted for exposure to LGBT violence when they present themselves as TGD or in gender-nonconforming ways. Our findings may not be representative of all youths because our sample was recruited in part from SGM-focused community organizations. It could be that youths involved in these groups have a more positive sexual and gender identity development and receive more support in general. Additionally, the data from this study are more than a decade old, and much has changed in prevalence and visibility, care and affirmation, and political debates regarding TGD youths.^60^ New prospective, community-based studies are needed to understand gender identity development and change, and associations with mental health for cisgender and TGD youths.

## Conclusion

This cohort study documented a diversity of gender identity trajectories in adolescence and early adulthood. Changes in gender identity were not associated with depressive symptoms. Furthermore, the group of youths who reported the most change in gender identities were among those with the lowest level of depressive symptoms. These findings suggest a pattern in which gender identity exploration is a normal part of adolescent development for some youths.^48,52^ Acknowledgment of this by health care clinicians^22^ may help alleviate anxiety related to treating TGD youths whose gender identity may change across time. Although youths who changed from a cisgender to a TGD identity reported higher levels of depressive symptoms at baseline compared with consistently cisgender youths, these differences were explained by higher exposure to LGBT violence. Health care clinicians should pay particular attention to youths transitioning to TGD identities; additional support in this process can help mitigate the adverse effects of exposure to LGBT violence from peers or family rejection. Moreover, delays in providing care can result in more stress for these youths.^61^ Ultimately, more longitudinal studies are needed to understand gender identity trajectories and health.

## Notes

Supplement 1. **eFigure.** Flowchart of Participation in the Study: Final Analytic Sample **eAppendix 1.** Additional Details About Participants and Procedures **eAppendix 2.** Detailed Information on Measures **eTable.** Sensitivity Analysis

Supplement 2.
Data Sharing Statement
