# Error in Citation

**DOI:** 10.1001/jamanetworkopen.2024.23707

**Published:** 2024-06-20

In the Original Investigation titled “Trajectories of Gender Identity and Depressive Symptoms in Youths,”^1^ published May 22, 2024, there was an error for an author’s name in the citation and author contributions of the article, which should have appeared as Real AG in the citation and Real in the author contributions. This article has been corrected.^1^
