# Supplementary material for: Trajectories of Gender Identity and Depressive Symptoms in Youths
Source: JAMA Netw Open. 2024 May 22;7(5):e2411322. doi: 10.1001/jamanetworkopen.2024.11322 (PMC11112442; doi:10.1001/jamanetworkopen.2024.11322)
Supplement: Supplement 1. — eFigure. Flowchart of Participation in the Study: Final Analytic Sample eAppendix 1. Additional Details About Participants and Procedures eAppendix 2. Detailed Information on Measures eTable. Sensitivity Analysis [file jamanetwopen-e2411322-s001.pdf]

Supplemental Online Content

Real AG, Lobato MIR, Russell ST. Trajectories of gender identity and depressive symptoms in youth. *JAMA Netw Open.* 2024;7(5):e2411322. doi:10.1001/jamanetworkopen.2024.11322

eFigure. Flowchart of Participation in the Study: Final Analytic Sample

eAppendix 1. Additional Details About Participants and Procedures

eAppendix 2. Detailed Information on Measures

eTable. Sensitivity Analysis

This supplemental material has been provided by the authors to give readers additional information about their work.

© 2024 Gonzales Real et al. *JAMA Network Open.*

eFigure. Flowchart of Participation in the Study: Final Analytic Sample

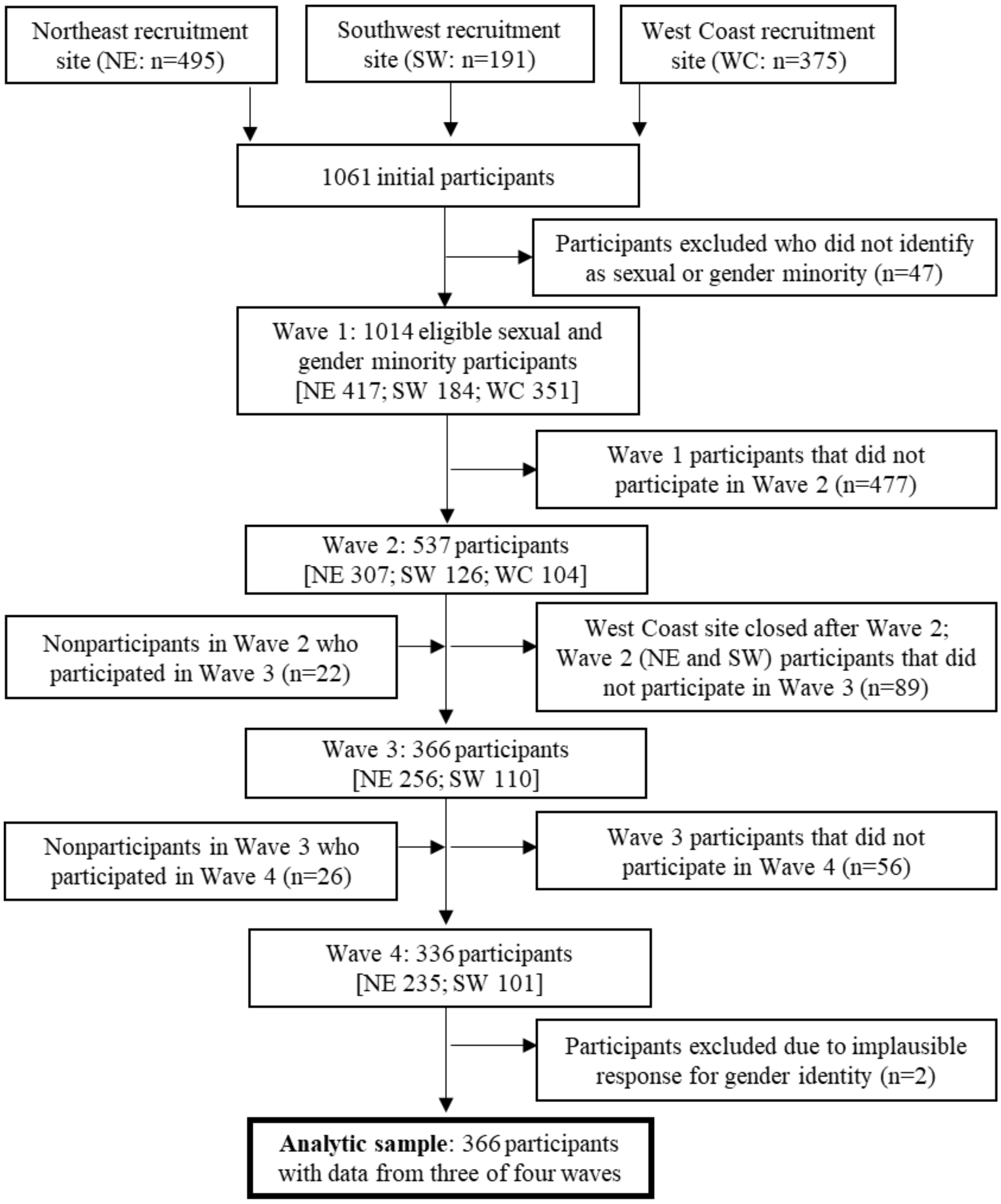

© 2024 Gonzales Real et al. *JAMA Network Open.*

eAppendix 1. Additional Details About Participants and Procedures

Although data collection occurred in three urban cities in the United States (one in the Northeast, one in the Southwest, and one in the West Coast), data presented in this study do not include participants from the West Coast site because the site was closed after the first two waves of data due to high attrition. Analyses to examine gender identity variability focus on participants who had data for at least three waves of the study.

Wave 1 was collected between November/2011 and October/2012, Wave 2 was collected between August/2012 and October/2013, Wave 3 was collected between July 2013 and August/2014, and Wave 4 was collected between June/2014 and June/2015.

Response rates based on the Wave 1 eligible sample across waves were 53% at Wave 2 (n = 537), 36% at Wave 3 (n = 366), and 33% at Wave 4 (n = 336).

© 2024 Gonzales Real et al. *JAMA Network Open.*

eAppendix 2. Detailed Information on Measures

Cumulative experiences of violence towards LGBT identity

Items had a four-point scale that ranged from 0 (*never*) to 3 (*at least three times*) and explored experiences of verbal insults, threats, and physical and sexual violence. For example, at Wave 1 participants were asked: “In your lifetime, how often have any of the following things happened to you because of your sexual orientation or identity or because people think you are lesbian, gay, bisexual, or transgender?”. Then, participants reported how often they had experienced six different experiences of LGBT violence: “verbal insults”, “threats of physical violence”, “objects thrown at you”, “punched, kicked or beaten”, “threats with a knife/gun/other weapon”, and “sexually attacked/raped”. For the following waves, participants reported experiences of LGBT violence considering the past nine months.

Demographic covariates

Receipt of free lunch in high school was included in our analysis as a proxy variable for socioeconomic status. Race and ethnicity was examined by asking the following: 1) “What is your ethnicity?”; and 2) “What is your race?”. Participants who were Latino/Hispanic were coded as Latino, regardless of race. Non-Latino participants were coded based on their reported race (e.g., Non-Latino White, Non-Latino Black). Participants who selected Asian, Pacific Islander, Native American, Multiracial or who did not report a race were coded as “Another race/Not reported”.

© 2024 Gonzales Real et al. *JAMA Network Open.*

eTable. Sensitivity Analysis

Multivariate Hierarchic Linear Model of Predictors of Depression not including participants who had missing in the cumulative exposure to LGBT violence (*N* = 355)			
	**Model 3**		
	b	***SE***	***p***
**Fixed Effects**			
Within-person (Level-1)			
Linear Time Change*	-0.67	(0.26)	0.01*
Cumulative exposure to LGBT violence	1.54	(0.69)	0.03*
Frequency of gender identity variability	0.41	(0.73)	0.58
Between-person (Level-2)			
Gender Identity Trajectory Membership[a]			
TGD	2.61	(2.38)	0.27
CIS→TGD	2.32	(2.39)	0.33
TGD→CIS	-3.21	(2.47)	0.20
Time x Gender identity trajectory membership[a]			
Linear time change x TGD	-0.69	(0.74)	0.35
Linear time change x CIS→TGD	0.29	(0.81)	0.72
Linear time change x TGD→CIS	0.19	(0.78)	0.81
Receipt of Free Lunch [b]			
Yes	-0.47	(0.97)	0.63
Not Reported	-10.04	(6.38)	0.12
Age (Centered at the mean)	-0.57	(0.28)	0.04*
Sex Assigned at Birth - Female [c]	3.80	(0.95)	<.001***
Recruitment Site - Southwest [d]	2.00	(1.02)	0.05
Race/ethnicity [e]			
Non-Latino Black	-3.87	(1.45)	0.008*
Latino	-1.49	(1.27)	0.24
Other/not reported	-1.97	(1.53)	0.20
Hormone use - Yes[b]	-4.23	(2.75)	0.12
Puberty blocker use - Yes[b]	-4.23	(2.75)	0.12
Cumulative exposure to LGBT violence	2.43	(0.52)	<.001***
Frequency of gender identity variability	2.60	(2.52)	0.30
Intercept	11.90	(1.38)	
**Random effects**			
Time variance	6.69	(1.23)	
Intercept variance	69.12	(7.47)	
Time and intercept covariance	-8.17	(2.44)	
Residual variance	37.65	(2.11)	

Notes: *, p<.05, ** p<.01, *** p<.001. SE = Standard Error.

* Slope of depressive symptoms for the CIS group

[a] Reference group = CIS

[b] Reference group = No

[c] Reference group = Male

[d] Reference group = Northeast

[e] Reference group = Non-Latino White

© 2024 Gonzales Real et al. *JAMA Network Open.*
