# Supplementary material for: Trajectories of Gender Identity and Depressive Symptoms in Youths
Source: JAMA Netw Open. 2024 May 22;7(5):e2411322. doi: 10.1001/jamanetworkopen.2024.11322 (PMC11112442; doi:10.1001/jamanetworkopen.2024.11322)
Supplement: Supplement 2. — Data Sharing Statement [file jamanetwopen-e2411322-s002.pdf]

Data Sharing Statement

Gonzales Real. Trajectories of Gender Identity and Depressive Symptoms in Youths. *JAMA Netw Open*. Published May 22, 2024. doi:10.1001/jamanetworkopen.2024.11322

Data
Data available: No
